# Hydrological drought trends and seasonality in selected Polish catchments between 1993 and 2022 using a threshold based approach

**DOI:** 10.1038/s41598-025-24133-1

**Published:** 2025-11-18

**Authors:** Ewelina Janicka-Kubiak

**Affiliations:** Department of Land Improvement, Environmental Development and Spatial Management, Poznań University of Life Sciences, Piątkowska 94, Poznań, 60-649 Poland

**Keywords:** Streamflow drought, Hydrological values, Temporal variability, Land use change, Hurst exponent, Climate sciences, Environmental sciences, Hydrology, Water resources

## Abstract

Ongoing climate change and Land Use Change (LUC) in Europe are altering the frequency and seasonality of low-flow events. Increases in mean annual temperature, shifts in catchment land use, and reduced retention capacity contribute to intensifying streamflow deficits. This study examines long-term trends and seasonality of hydrological droughts in selected catchments of the Central European Lowland in Poland over hydrological years 1993–2022. Low-flow events were identified and characterised using tools in the R programming environment. Trends were assessed with the Mann–Kendall (MK) test and Sen’s slope estimator, and persistence was evaluated with the Hurst exponent. Significant correlations (*p* < 0.05) were found between the Total Number of Drought Events (TNDE) and LUC, particularly for events lasting ≥ 7 days at Q90 threshold, with the strongest effects in the Skora, Luciąża, Widawka and Grabia rivers. A marked increase in summer and autumn low-flow events was recorded after 2002, alongside rising Seasonal Number of Drought Days (SNDD) and Seasonal Cumulative Deficit Volume (SCDV), reflecting growing water deficits. The persistence of these trends suggests continued streamflow decline. The findings can inform the identification of catchments vulnerable to worsening summer water conditions.

**Supplementary Information:**

The online version contains supplementary material available at 10.1038/s41598-025-24133-1.

## Introduction

 Drought is a complex and recurrent natural hazard that affects ecosystems and societies across diverse temporal and spatial scales^1–3^. It has both environmental and socio-economic impacts, making it one of the most impactful natural disasters. Droughts result from precipitation deficits of considerable magnitude, persisting over extended periods. Because these deficits propagate through various stages of the hydrological cycle, four main types of droughts are commonly distinguished: meteorological, agricultural, hydrological, and socio-economic droughts^4^.

The occurrence and severity of drought are strongly influenced by two key factors: climate change and LUC^5^. Moreover, the combined effects of natural processes and human activity are the primary drivers of changes in climatic extremes and the resulting shifts in hydrological extremes^6^. According to Beran and Rodier^7^ and the Flow Regimes from International Experimental and Network Data (FRIEND) research programme^4^, drought is understood as a sustained, regionally extensive period of below-average natural water availability, whether in precipitation, runoff, or groundwater. Human activities and the resultant global warming have been identified as the main causes of the increasing frequency and intensity of drought events^8^. Globally, drought frequency and intensity are expected to increase, particularly in the Northern Hemisphere, with regions, such as the USA, China, India, South Korea, Vietnam, and Europe projected to experience worsening conditions^5,9–14^.

Both climate change and LUC are known to pose risks to water resources^15^. Climate change has intensified extreme hydro-climatic events, and it is likely to continue increasing their frequency and intensity in the near future^16,17^. In recent decades, global warming has been well documented, with the mean global temperature rising by 1.19 ± 0.12 °C above pre-industrial levels^18,19^, and further increases are anticipated^20^. These shifts are expected to have a huge impact on hydrological processes^21^.

At present, one of the principal tasks of hydrology is to identify regions vulnerable to the adverse effects of prolonged drought arising from climatic, hydrological, and hydrogeological conditions. To this end, it is essential to establish uniform criteria for distinguishing hydrological drought periods within long-term data series and to quantify this phenomenon^22^. Poland is among the regions highly vulnerable to severe hydrological droughts^23^. The areas most susceptible to atmospheric drought in Poland are the Central Polish Lowlands and the western part of the Pomeranian Lakeland^24,25^. Records of river low-flows have long been kept, with the earliest references to exceptionally low water levels in Polish territories dating back to 988, 1121, 1332, and 1473 ^26^. Severe hydrological droughts affecting the whole of Poland occurred in the first half of the 1950 s, the early 1960 s, the mid-1980s, the early 1990 s, the mid-2000s, and the early 2010 s, indicating that Poland experiences significant water shortages on average every 10 to 15 years; however, prolonged low-flows with substantial streamflow deficits across all rivers simultaneously have been a rare phenomenon^23^. Poland has experienced several severe hydrological droughts over the last few decades, notably in 1992–1993, 2006 and 2008. In both 2012 and 2015, record low water levels of the River Vistula in the capital, Warsaw, were widely reported in the Polish media^27^. The year 2022 was considered particularly catastrophic in Europe in terms of hydrological drought. During the summer of 2022, Central and Southern Europe experienced an extreme drought, characterised by exceptionally low soil moisture and river water levels, severely affecting sectors across many countries^28^.

Processes occurring on the Earth’s surface influence the intensity of extreme events, such as heat waves and droughts. Land transformation in mid-latitude regions has significantly increased the frequency of hot, dry summers^29^. Human-induced climate change leads to an accelerated water cycle and a more variable climate, which in turn results in greater fluctuations in river discharge^30^. Hydrological droughts, characterised by gradual onset and prolonged duration, result in substantial human and economic losses, with wide-ranging impacts^31,32^. In recent decades, severe droughts have affected nearly every continent^33–35^.

Notwithstanding the findings from earlier studies it remains unclear which causes of extreme hydrological events are decisive. Although the impact of climate change on droughts is well-documented, the effects of LUC are less understood. Recent research has identified climate change as the primary driver behind the projected increase in drought frequency^36,37^. However, there is limited information on hydrological changes in smaller catchments, which can substantially influence entire river basins. In smaller catchments, low-flow periods are more evenly distributed over time, with hydrological droughts in the 1960 s and 1970 s affecting mountain rivers more severely, while those of the 1980 s and 1990 s had a greater impact on lowland catchments^23^. Small rivers play a crucial role in regulating basin-wide water flow and quality, acting as natural buffers for groundwater recharge. Their condition directly affects the stability of the wider basin, contributing to nutrient cycling, supporting biodiversity, and serving as critical water sources for agricultural and ecological systems. In lowland river basins dominated by agriculture, water availability for plant growth is particularly important^28^. In lowland areas, the duration of low-flows depends on basin water resources and the direction of water management operations, yet severe low-flow periods do not necessarily correspond proportionally to preceding ones^23^. Consequently, alterations in small river systems may exert substantial influence on the hydrological stability and functioning of the entire basin^38,39^.

There is a wealth of information in the literature on hydrological drought prediction for future years based on hydrological models^40,41^. For monitoring droughts, numerous typical drought indices have been developed based on meteorological and hydrological variables to assess the magnitude, duration, severity, and spatial extent of droughts. One example is the Hurst exponent (H), first introduced by Hurst^42^ in his analysis of Nile River floods. Since then, this exponent has been applied in many scientific disciplines. Recent research on Hurst behavior in climate and hydrology is reported, for example, by O’Connell^43^ and Adarsh and Priya^44^. In this study, we used the Hurst exponent to evaluate the persistence of drought trends and their potential continuation in the study area.

However, there is a lack of comprehensive studies, focused on small lowland catchments that exert a substantial influence on larger basins.

**Fig. 1:**
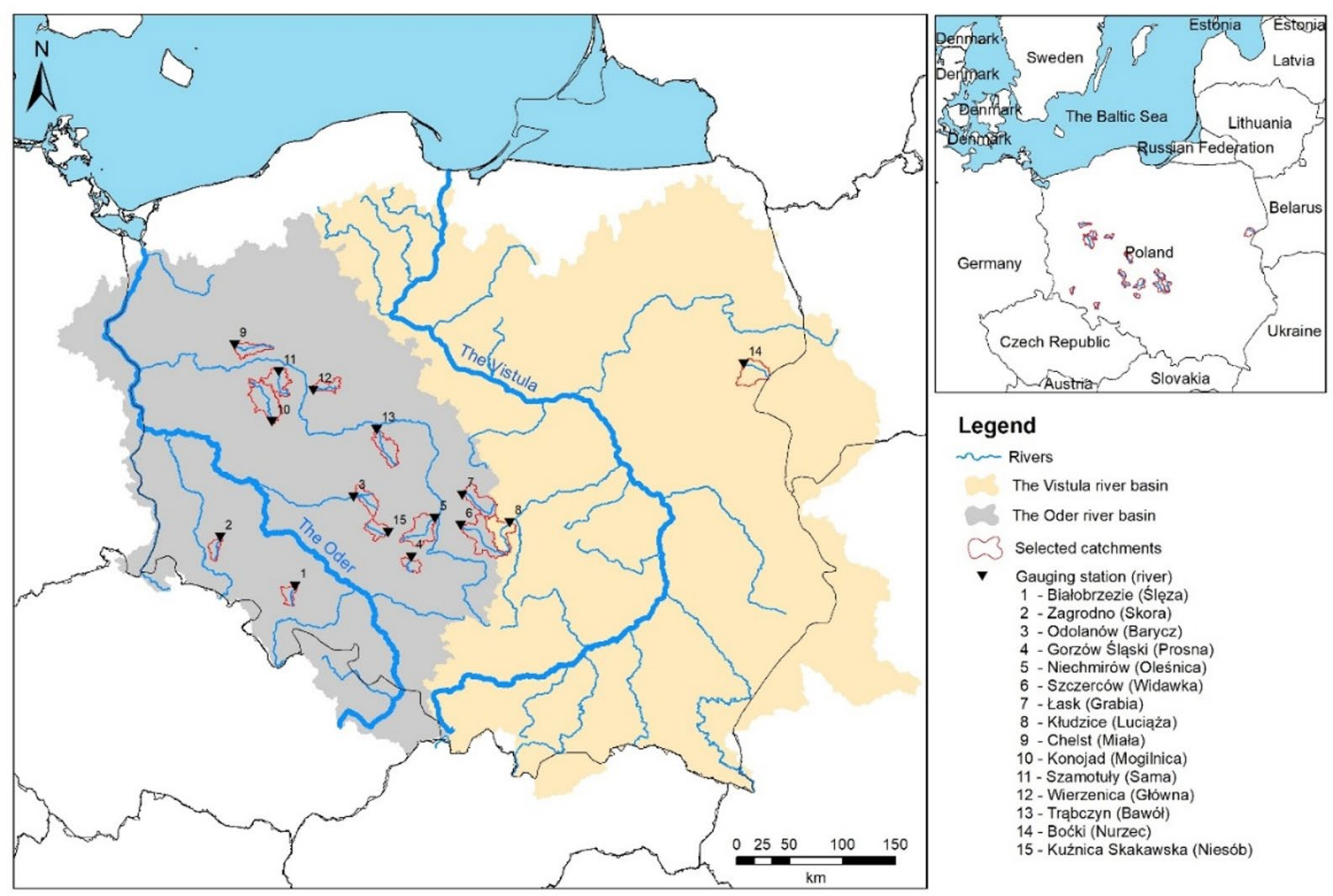
Location of selected catchments.

This study aims to address that knowledge gap through a case study of several rivers located in the Polish lowlands (Fig. 1). Societal welfare and economic development in a region largely depend on effective integrated water resources management at catchment scale, as well as the implementation of drought monitoring and preparedness strategies. Accordingly, there is a growing need to further analyse hydrological droughts and their characteristics.

In this paper, we present a comprehensive drought analysis that involves identifying drought events and characterising their properties, including duration, intensity, cumulative deficit volume, and spatial extent. We also examine indicators related to drought seasonality and apply the peaks-over threshold (POT) method. Over the years, many indices have been proposed based on factors, such as the nature of the water deficit, temporal resolution, regionalisation, or standardisation. The most widely used approach for identifying drought periods is the POT method, in which a drought is defined as a period when river flow falls below a specified threshold (Qt). This method, known as the threshold approach, was introduced by Yevjevich in the United States^45^ and by Zielińska in Poland^46^. In this study, the POT method is applied to selected cross-sections, where low-flow events are analysed using three thresholds that correspond to different drought types. It is assumed that Qt = Qp, meaning the threshold flow corresponds to a selected percentile of flow exceedance. The most commonly used thresholds are Q70 and Q90^47–49^, while Tallaksen and van Lanen^4^ recommend using thresholds from Q70 to Q95. In Poland, a commonly recommended methodology includes three thresholds: Q70, Q90, and Q95^50^. Moderate drought is defined by the Q70 flow, which serves as a warning level; Q90 defines deep drought, considered a state of emergency; and Q95 represents extreme drought, typically classified as natural disasters. Considering these thresholds, the study aims to comprehensively analyse water deficits under all three variants and to evaluate the effects of land use changes within the analysed catchments. An additional objective is to calculate the Hurst exponent in order to evaluate the persistence of flow patterns and identify potential future trends in water availability.

## Results

### Catchments

The MK test was performed for 15 analysed rivers. The MK test indicated no statistically significant trend for the Nurzec River). Additionally, an increasing trend was identified for the Niesób River. Based on these findings, both rivers were excluded from further analysis. The Sen’s slope estimator confirmed the increasing trend for the Niesób River. In contrast, the remaining rivers exhibited negative slope values, indicating a decline in river flows over time. Notably, the Skora River showed the lowest Sen’s slope value, suggesting the most pronounced negative trend in water flow among the rivers studied (Table 1).

**Table 1:** Hydrographical characteristics of selected rivers and gauging stations and significance and direction of trends in time series (1993–2022) based on the MK test. The direction of the trend is indicated by the sign of the sen’s slope estimator (+ or −).

No.	River	Gauging station	Area [km^2^]	Q_min (long−term)_ [m^3^s-^1^]	Q_mean (long−term)_ [m^3^s-^1^]	Q_max (long−term)_ [m^3^s-^1^]	MK test	*p*	Sen’s Slope
1	Ślęza	Białobrzezie	176.93	0.004	0.440	16.300	-	4.6E-07	−3.25E-06
2	Skora	Zagrodno	165.72	0.060	0.776	33.800	-	< 2.2e-16	−6.08E + 06
3	Barycz	Odolanów	310.27	0.020	0.773	18.100	-	< 2.2e-16	−2.29E-05
4	Prosna	Gorzów Śląski	176.10	0.090	0.751	27.100	-	< 2.2e-16	−2.35E-05
5	Oleśnica	Niechmirów	601.69	0.250	2.260	58.200	-	< 2.2e-16	−4.43E-05
6	Widawka	Szczerców	726.28	1.010	4.555	26.100	-	< 2.2e-16	−1.73E-04
7	Grabia	Łask	473.04	0.270	2.444	44.200	-	< 2.2e-17	−4.60E-05
8	Luciąża	Kłudzice	547.88	0.140	2.699	43.500	-	3.3E-10	−2.24E-05
9	Miała	Chełst	301.92	0.160	1.230	3.730	-	< 2.2e-16	−3.53E-05
10	Mogilnica	Konojad	705.03	0.004	1.492	18.400	-	< 2.2e-17	−3.77E-05
11	Sama	Szamotuły	397.41	0.004	0.875	10.500	-	< 2.2e-16	−3.24E-05
12	Główna	Wierzenica	213.89	0.033	0.545	6.080	-	< 2.2e-17	−2.97E-05
13	Bawół	Trąbczyn	429.04	0.000	1.350	19.500	-	< 2.2e-16	−2.48E-05
14	Nurzec	Boćki	516.06	0.120	2.105	45.100	-	6.1E-02	−5.73E-06
15	Niesób	Kuźnica Skakawska	259.87	0.049	0.950	12.200	+	2.5E-08	7.01E-06

**Fig. 2:**
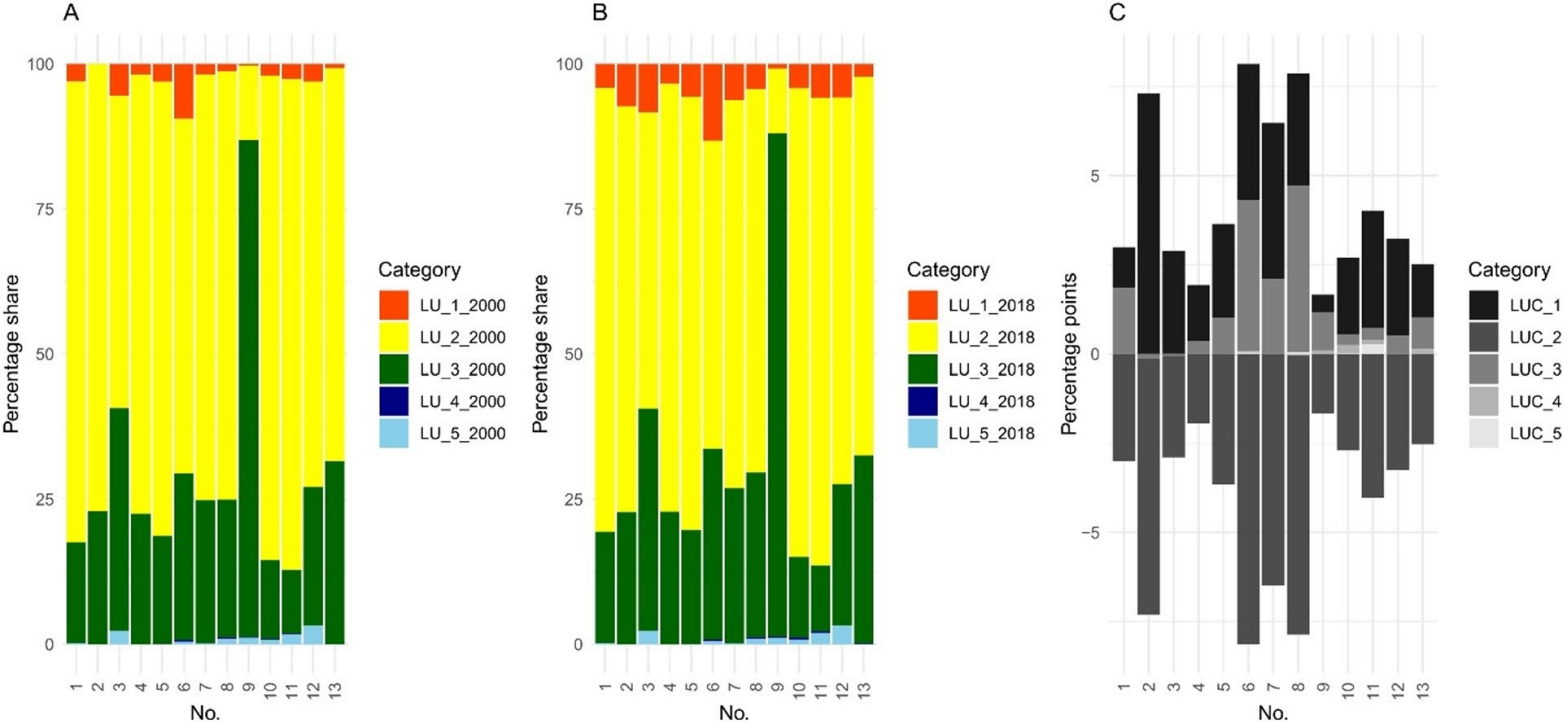
Land use (LU) in 2000 (**A**) and 2018 (**B**), and LUC (**C**) for selected catchments. Legend: 1 – artificial surfaces; 2 – agricultural areas; 3 – forest and semi-natural areas; 4 – wetlands; 5 – water bodies.

Based on the 2018 CORINE Land Cover data (CLC), agricultural land dominates the analysed catchments, covering on average more than 64% of the area (Fig. 2). The Sama River catchment has the highest proportion of agricultural land at 80.6%. In contrast, the Miała River catchment is predominantly forested, with forested land accounting for 86.7% of its area. The catchment with the highest percentage of anthropogenic land use is the Widawka River catchment, where built-up areas represent 13.3% of the total surface (see Supplementary Fig. S1 online). An analysis of land cover changes between 2000 and 2018 shows that all catchments underwent measurable transformation. During this period, the percentage of anthropogenic land increased in all catchments, with an average gain of 2.58% points (pp). The largest increase was observed in the Skora River catchment, where the share of built-up areas rose by 7.3 pp at the expense of agricultural and forest land. Notable changes were also recorded in the Sama, Luciąża, Grabia, and Widawka catchments, where the share of anthropogenic land increased by 3.9–4.3 pp.

The analysed catchments were also classified according to soil types. The predominant soils are podzolic (PZ), which are highly permeable and cover an average of 36% of the area, and luvisols (LV), which are poorly permeable and account for approximately 38% of the area. The Miała River catchment lies almost entirely (97%) within areas dominated by PZ, indicating high permeability. In contrast, the Skora River catchment is primarily composed of LV (see Supplementary Fig. S2 online).

### Streamflow drought

The analysis of hydrological data for selected rivers during the hydrological years 1999–2022 allowed for the assessment of total drought duration for periods of at least 7 days, classified into three levels: moderate (Q70), deep (Q90), and extreme (Q95) (Table 2).

The rivers with the highest number of flow drought events (126, 115, and 112) are: the Ślęza River, the Skora River, and the Prosna River, respectively. The pattern of fewer, drought periods in rivers (i.e. Sama River and Bawół River) where TNDE was less than 50 for Q70 and lasted at least 7 days, can be attributed to the geological morphology (dominance of well-drained soils, such as FL, PZ and CM). In the case of the Ślęza River where the highest TNDE values were recorded for Q70 flow and minimum 7-day duration, extending the drought period to minimum 30 days resulted in only 28 events with flow limit value Q70, 8 events with limit value Q90, and 4 events with Q95. However, in rivers where low TNDE values were observed (for the threshold value of Q70 and duration of ≥ 7 days), such as the Grabia River and the Sama River, extending the minimum low-flow duration to ≥ 30 days led to higher TNDE values across all threshold flow variants compared to the other analysed rivers. Analysis of drought events lasting at least 20 and 30 days reveals less frequent but more persistent periods of streamflow deficit, which is of key importance for assessing the risk of prolonged water shortages in water management. (Tables 2 and 3). This suggests that in catchments with a high TNDE, drought episodes tend to be short and frequent, while in catchments with fewer events, the episodes are generally longer and more persistent, which indicates that drought periods are rare but long-lasting. Furthermore, as drought duration extends, the Mean Cumulative Deficit Volume (MCDV) increases across all rivers, with the highest MCDV observed in the Sama River during a moderate drought (Q70) lasting at least 7 days. The highest Mean Cumulative Deficit Volume (MCDV) was observed for the Ślęza River under the Q70 threshold for drought durations of at least 30 days (Table 4).

**Table 2:** Hydrological values of the TNDE for the Q70, Q90, Q95 threshold levels at gauging stations on selected rivers (1993–2022).

No.	TNDE for river	Duration ≥ 7 d			Duration ≥ 10 d			Duration ≥ 20 d			Duration ≥ 30 d		
		Q70	Q90	Q95	Q70	Q90	Q95	Q70	Q90	Q95	Q70	Q90	Q95
1	Ślęza	126	40	21	99	27	17	44	13	9	28	9	4
2	Skora	115	44	22	87	30	12	39	11	4	21	4	2
3	Barycz	87	42	23	80	32	15	45	12	7	35	7	3
4	Prosna	112	27	18	88	20	17	49	12	6	31	6	4
5	Oleśnica	64	31	13	50	28	10	36	18	5	28	8	3
6	Widawka	69	31	19	61	29	15	42	20	8	32	11	4
7	Grabia	59	32	15	50	27	13	39	17	8	32	14	6
8	Luciąża	95	42	25	78	33	20	48	17	4	32	6	2
9	Miała	75	35	17	58	25	14	38	12	9	30	8	6
10	Mogilnica	52	21	22	46	18	15	34	15	10	28	12	7
11	Sama	45	26	13	39	24	11	38	16	9	31	13	6
12	Główna	67	23	13	50	20	12	33	15	3	27	9	3
13	Bawół	49	21	15	46	20	12	31	16	9	28	12	5

**Table 3:** Hydrological values of the MDD for the Q70, Q90, Q95 threshold levels at gauging stations on selected rivers (1993–2022).

No.	MDD for river	Duration ≥ 7 d			Duration ≥ 10 d			Duration ≥ 20 d			Duration ≥ 30 d		
		Q70	Q90	Q95	Q70	Q90	Q95	Q70	Q90	Q95	Q70	Q90	Q95
1	Ślęza	22.43	21.58	21.24	26.36	28.19	24.29	42.66	43.92	33.67	53.50	52.56	47.75
2	Skora	20.99	17.07	15.82	24.49	21.43	22.25	38.73	34.36	37.25	55.87	50.75	51.00
3	Barycz	33.91	22.64	19.22	36.18	27.19	25.20	53.00	47.58	37.29	61.43	64.57	56.33
4	Prosna	26.08	28.15	21.72	31.03	34.95	22.53	45.06	50.25	41.33	57.42	76.00	51.25
5	Oleśnica	47.75	33.68	32.92	58.96	36.43	40.50	76.53	48.83	66.00	92.07	78.88	95.33
6	Widawka	44.59	31.13	24.21	49.41	32.72	28.47	66.07	41.60	38.75	79.06	56.27	54.75
7	Grabia	51.62	30.53	33.00	59.15	34.85	36.77	72.78	47.12	51.00	84.66	51.36	59.50
8	Luciąża	31.17	19.69	14.56	36.18	22.91	16.25	49.54	32.00	33.00	61.69	46.33	39.00
9	Miała	38.42	21.40	23.12	46.83	26.84	26.50	64.39	40.58	35.11	75.23	48.63	39.83
10	Mogilnica	57.56	47.90	22.27	64.83	54.67	29.07	83.77	63.13	36.70	95.97	72.67	42.00
11	Sama	68.80	34.00	32.46	78.21	36.17	36.91	79.92	46.56	41.89	92.19	51.69	50.67
12	Główna	42.97	33.17	25.85	55.02	36.90	27.25	76.18	44.93	72.33	87.59	59.22	72.33
13	Bawół	65.02	44.67	34.13	68.72	46.55	40.67	95.39	54.63	49.33	103.07	65.42	69.60

**Table 4:** Hydrological values of the MCDV for the Q70, Q90, and Q95 threshold levels at gauging stations on selected rivers (1993–2022).

No.	MCDV for river	Duration ≥ 7 d			Duration ≥ 10 d			Duration ≥ 20 d			Duration ≥ 30 d		
		Q70	Q90	Q95	Q70	Q90	Q95	Q70	Q90	Q95	Q70	Q90	Q95
1	Ślęza	4.92	1.68	1.00	6.26	2.49	1.23	14.09	5.17	2.33	22.14	7.47	5.24
2	Skora	3.51	1.78	1.31	4.64	2.62	2.40	10.34	7.13	7.20	19.20	19.62	14.40
3	Barycz	6.62	2.21	1.15	7.20	2.90	1.77	12.79	7.72	3.79	16.45	13.24	8.85
4	Prosna	4.04	2.77	1.74	5.14	3.74	1.84	9.22	6.24	5.22	14.58	12.47	7.83
5	Oleśnica	5.61	1.72	1.54	7.18	1.91	2.00	9.98	2.97	4.00	12.83	6.68	6.67
6	Widawka	6.05	2.39	1.41	6.85	2.56	1.78	9.94	3.71	3.34	13.05	6.74	6.69
7	Grabia	7.78	2.06	1.90	9.18	2.44	2.19	11.77	3.88	3.56	14.35	4.71	4.75
8	Luciąża	5.62	1.84	1.26	6.85	2.34	1.57	11.13	4.55	7.86	16.70	12.89	15.73
9	Miała	6.60	2.57	2.61	8.42	3.60	3.17	12.85	7.50	4.94	16.28	11.25	7.40
10	Mogilnica	6.95	2.15	0.53	7.86	2.51	0.78	10.63	3.01	1.18	12.91	3.77	1.68
11	Sama	9.98	1.65	1.22	11.51	1.79	1.44	11.82	2.69	1.76	14.49	3.31	2.64
12	Główna	6.24	1.79	1.12	8.36	2.05	1.21	12.67	2.74	4.84	15.48	4.56	4.84
13	Bawół	8.93	1.33	0.54	9.51	1.40	0.68	14.12	1.74	0.91	15.63	2.33	1.63

**Fig. 3:**
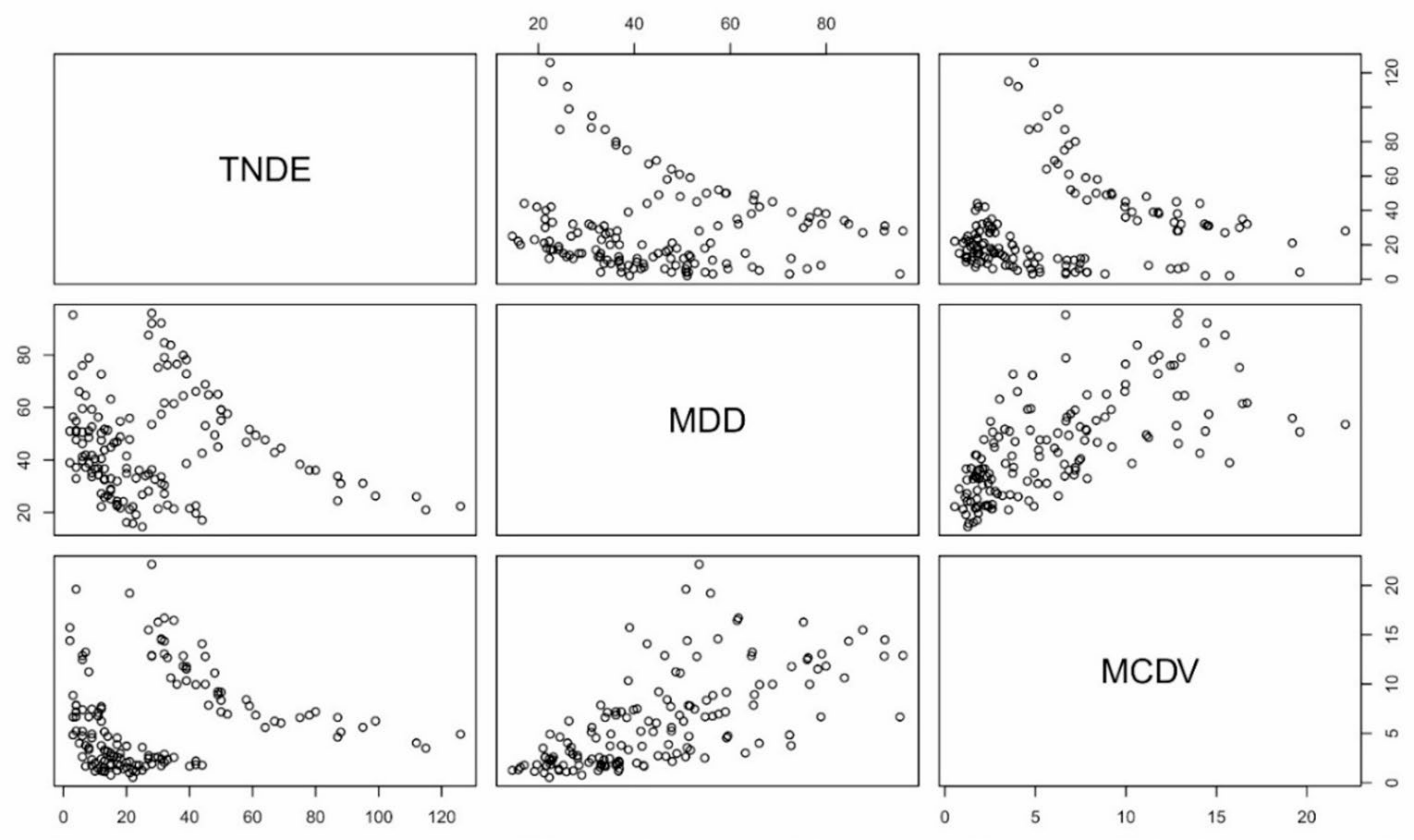
Correlation between the analysed parameters: TNDE, MDD, and MCDV (total for thresholds Q70, Q90, Q95).

A Student’s t-test revealed a statistically significant correlation between MDD and MCDV (*r* = 0.6521, *p* < 0.001), indicating a strong positive relationship. This analysis, performed collectively across all three examined percentiles, confirms an association between longer drought durations and greater cumulative water deficits. These findings indicate a heightened risk of water shortages and the occurrence of long-term droughts in the region (Fig. 3). Additionally, correlation analyses conducted separately for each percentile confirmed the overall observed relationship. The results demonstrated consistency across all cases, with statistically significant correlations between MDD and MCDV observed for each percentile. The correlation coefficients were *r* = 0.62 for Q70 (*p* < 0.05), *r* = 0.51 for Q90 (*p* < 0.05), and *r* = 0.48 for Q95 (*p* < 0.05).

**Fig. 4:**
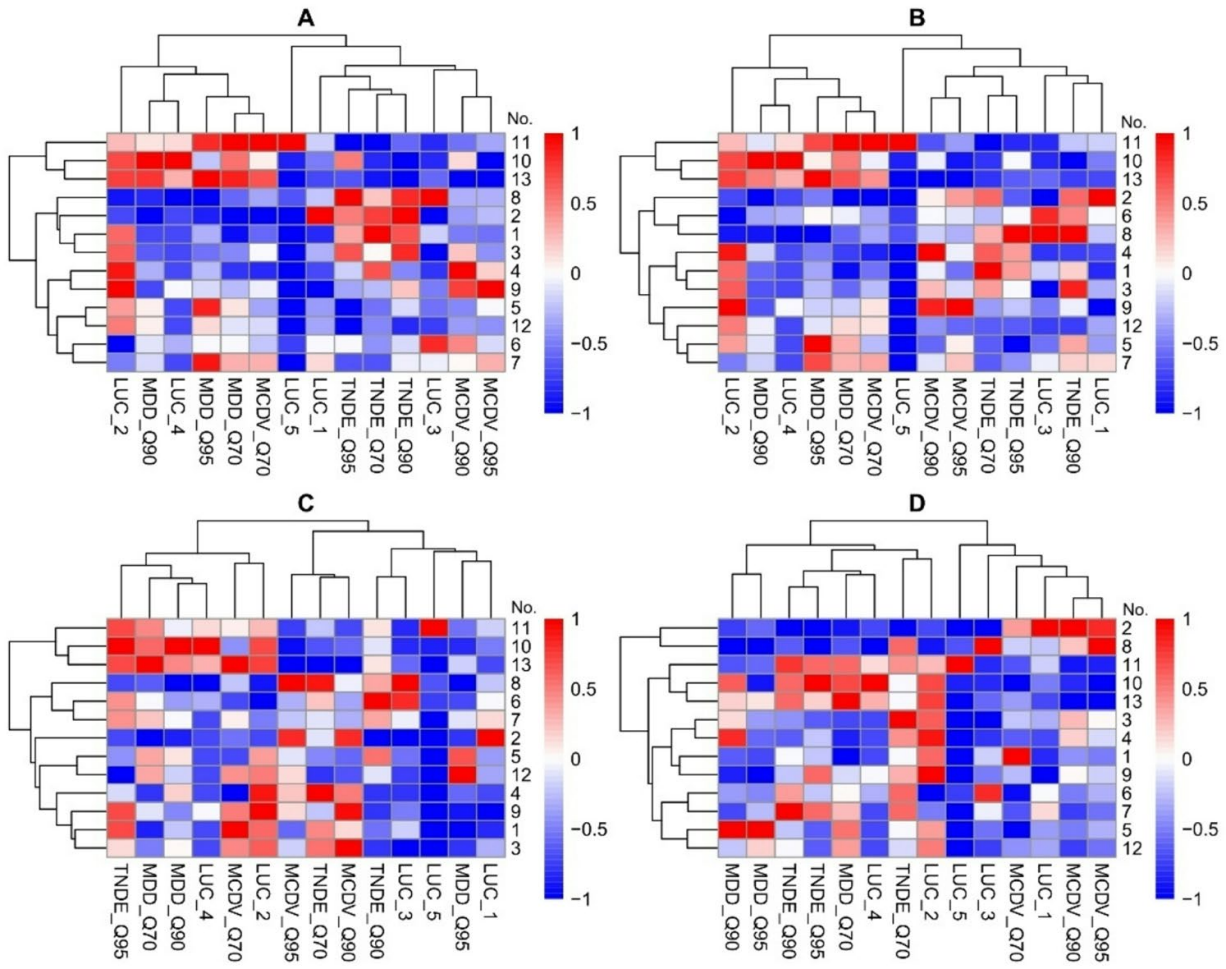
Heatmap with hierarchical clustering showing links between hydrological values and land use change (LUC) for selected rivers, based on drought durations of: A ≥ 7, B ≥ 10, C ≥ 20, and D ≥ 30 days.

The analysis of heat maps depicting the frequency of low-flow events of varying durations, in conjunction with land cover change data, enabled the identification of spatiotemporal patterns from a hydrological perspective (Fig. 4). The cluster analysis of standardised results allowed the identification of several groups of rivers based hydrological and LUC values, depending on drought duration. For droughts lasting at least 7 days, a group was identified including the Sama, Mogilnica, and Bawół rivers, which were characterised by the highest values of MDD_Q90, MDD_Q70, and MCDV_Q70. A second group comprised the Luciąża, Skora, Ślęza, and Barycz rivers, which exhibited the highest values of TNDE_Q70, TNDE_Q90, and TNDE_Q95, with the Skora River additionally showing a high value of the LUC_1 parameter. This suggests that land use changes, such as urbanisation and land surface sealing, considerably reduce infiltration and retention capacity, thereby increasing the frequency of short-term low-flow events. Extending the drought duration to at least 10 days, and then to 20 days, revealed that the Sama, Mogilnica, and Bawół rivers continued to exhibit high MDD_Q70 values, and for droughts of at least 20 days, high TNDE_Q95 values were also observed. For droughts lasting at least 30 days, the Skora River stood out with the highest MCDV_Q90, MCDV_Q95, and LUC_1 values, while the Sama, Mogilnica, and Bawół rivers continued to exhibit high TNDE_Q90, TNDE_Q95, and MDD_Q70 values. These findings imply that the most pronounced changes in the Skora River’s flow regime are associated with short-duration droughts, which are more susceptible to catchment-scale modifications affecting infiltration and water retention. Furthermore, the clustering of rivers into correlation classes shifted with increasing low-flow duration, suggesting that catchment responses vary not only in intensity but also in temporal behavior, reflecting spatial heterogeneity in their reaction to changing precipitation and flow conditions.

**Fig. 5:**
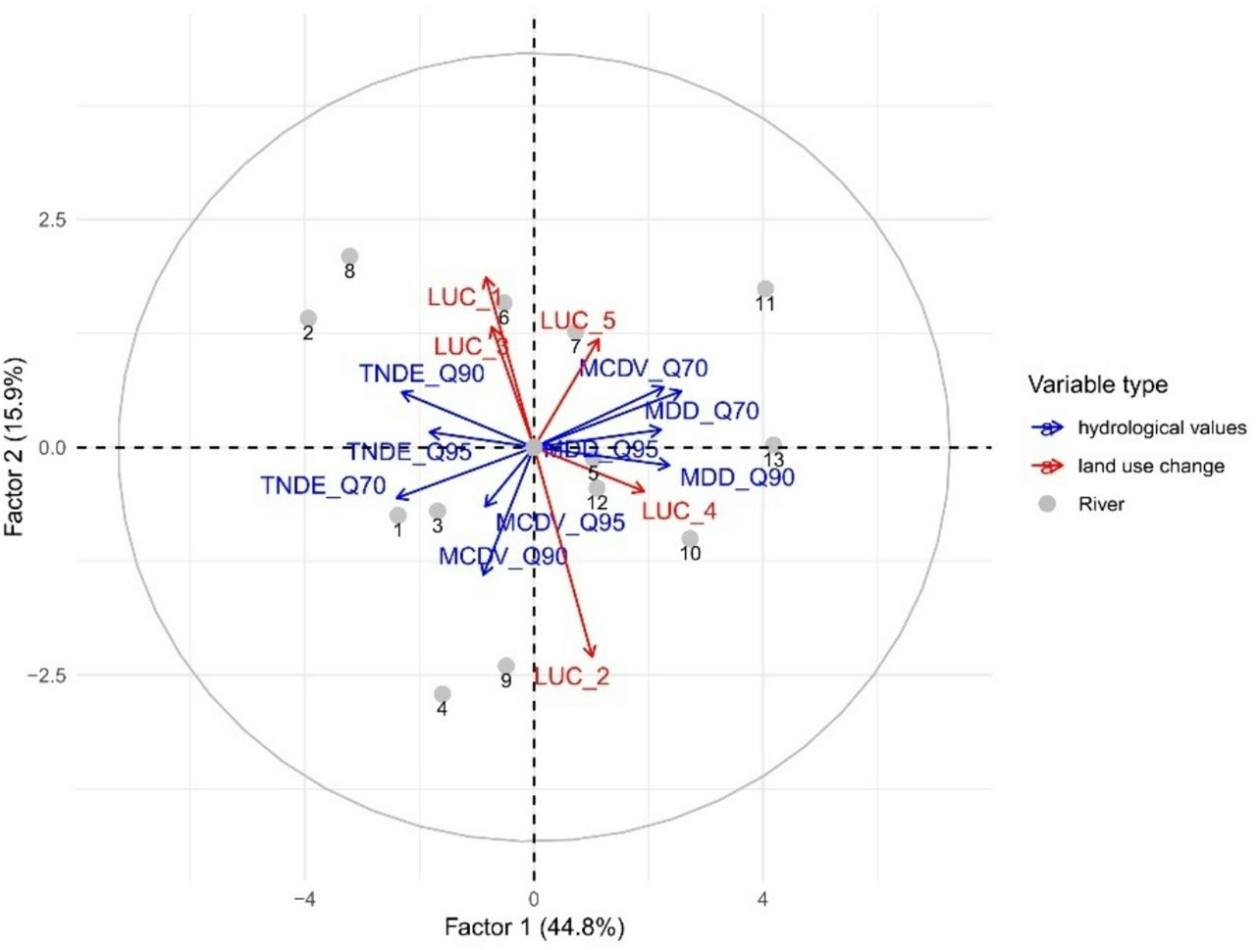
Principal Component Analysis (PCA) of hydrological parameter values and land use change for catchments.

The conducted Principal Component Analysis (PCA) explained 60.7% of the total variance associated with LUC and the analysed hydroclimatic indicators (TNDE, MDD, MCDV) for low-flow events lasting at least 7 days. This threshold was selected as it marks the shortest period indicative of hydrological drought onset. The first principal component accounted for 44.8% of the variability, while the second explained 15.9%. A strong negative correlation was observed between the increase in anthropogenic land cover (LUC_1) and the decrease in agricultural land area (LUC_2), indicating that urban expansion has occurred largely at the expense of arable land. Furthermore, LUC_1 showed a positive correlation with the TNDE_90 indicator, suggesting that the growth of built-up areas may contribute to an increased number of days affected by severe low-flow conditions, based on the 90th percentile threshold. This relationship was particularly evident in the catchments of the Skora, Luciąża, Widawka, and Grabia rivers, which have experienced considerable spatial transformation in recent years, marked by an increased proportion of built-up areas (Fig. 5).

The PCA performed separately for the years 2000 and 2018 revealed substantial changes in the LU_1 indicator (representing anthropogenic land cover) over the 18-year period. The results clearly show that by 2018, LU_1 became positively correlated with TNDE, indicating that the increase in human-modified land areas had begun to strongly influence the number of low-flow days. In contrast, in the 2000 analysis, LU_1 was a neutral factor with respect to TNDE (see Supplementary Fig. S3 online) This shift confirms the findings from the previously generated heat map, which also highlighted the growing impact of anthropogenic pressure on hydrological drought conditions.

A detailed seasonal drought analysis was conducted by dividing the year into four seasons (spring, summer, autumn, and winter) to capture temporal variability in low-flow events. Three hydrological drought indicators were calculated: Seasonal Number of Drought Days (SNDD), Seasonal Number of Drought Events (SNDE), and Seasonal Cumulative Deficit Volume (SCDV).

**Fig. 6:**
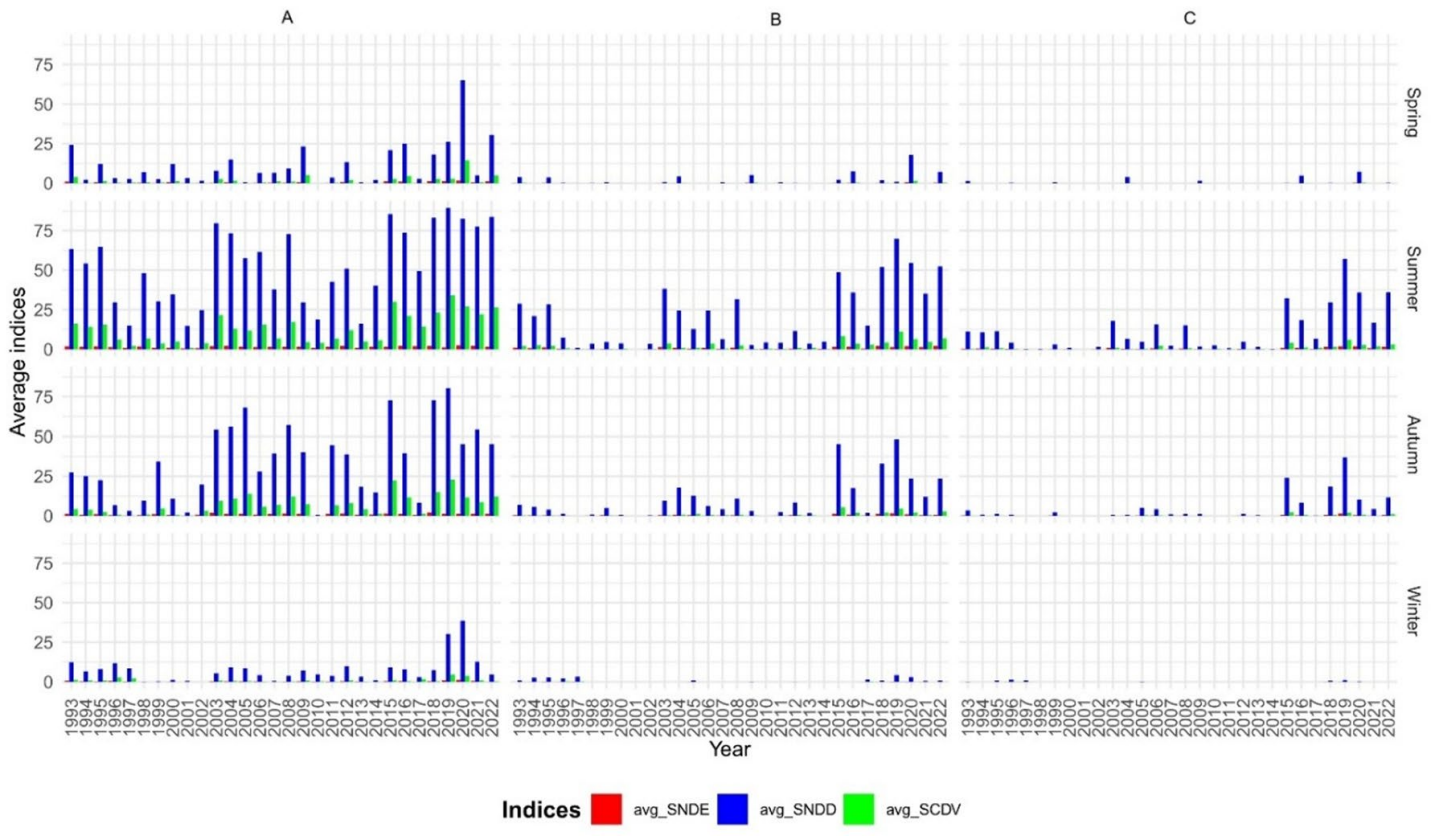
Average values of SDCV, SNDD and SNDE indices in the hydrological years 1993–2022 for different thresholds: (**A**) Q70, (**B**) Q90, (**C**) Q95, and in different seasons.

The analysis indicates a clear predominance of drought in summer and autumn across all percentile variants (Fig. 6). For the Q70 threshold, the mean SNDD reached 53 days in summer and 34 days in autumn. Under the Q95 threshold, which indicates extreme drought, these values dropped to 12 days in summer and just 1 day in autumn, suggesting that summer is the most drought-prone season in the studied region. Correlation analysis between the drought indices revealed statistically significant, strong relationships: SNDD and SNDE (*r* = 0.802, *p* < 0.05), and SNDD and SCDV (*r* = 0.702, *p* < 0.05). The strong correlation between SNDD and SNDE indicates that longer drought periods are associated with a greater number of drought events. Meanwhile, the positive correlation between SNDD and SCDV reflects an increasing water deficit in streamflow volumes during prolonged low-flow conditions, intensifying hydrological stress across catchments.

The Hurst exponent values for the analysed rivers ranged from 0.6844 to 0.8663, indicating strong autocorrelation and the persistence of trends in the flow time series (Table 5). This suggests that changes observed in successive years tend to follow the same directional pattern, pointing to the system’s so-called “hydrological memory”. The highest Hurst exponent values were recorded for the Główna and Miała rivers, suggesting that in these rivers, water flows are particularly influenced by past hydrological conditions and exhibit a high level of long-term predictability.

**Table 5:** Hurst exponent for the analysed rivers, based on data from the multi-year period 1993–2022.

No.	ID	Hurst exponent
1	Ślęza	0.7860
2	Skora	0.7155
3	Barycz	0.7925
4	Prosna	0.6844
5	Oleśnica	0.7735
6	Widawka	0.8077
7	Grabia	0.7351
8	Luciąża	0.7400
9	Miała	0.8663
10	Mogilnica	0.7995
11	Sama	0.8032
12	Główna	0.8431
13	Bawół	0.7571

## Discussion

Periods of low river flow have a considerable impact on ecosystems, the economy, and human life. Climate change is contributing to increasing challenges related to water availability, leading to a growing water deficit on a global scale. Trnka et al.^51^ highlight a rise in the frequency, duration, and severity of droughts in Central Europe, directly attributable to climate change. In addition, land use changes, such as urbanisation, deforestation, and agricultural intensification, disrupt the natural water cycle, reduce infiltration, and increase surface runoff, further exacerbating water scarcity^52^. The duration of low-flow periods is strongly influenced by water management activities in catchments subjected to intense anthropogenic pressure. One example of this is the dewatering of open-pit mines, which has caused low-flow conditions to persist for extended periods in catchments affected by depression cones^53^. At the global level, projected climate changes is expected to intensify the hydrological cycle, increasing the frequency and risk of hydrological extremes such as droughts and floods^54^. Monitoring drought conditions is therefore essential for mitigation, with the threshold level method^55^ being among the most widely applied approaches.

In recent years, Central and Southern Europe have experienced extreme drought characterised by exceptionally low soil moisture and river water levels. These events have had serious impacts across multiple sectors in several countries. Initially driven by a pronounced rainfall deficit, the meteorological drought eventually developed into low river flows with widespread effects^28^. By the middle of the century, both the frequency and intensity of heatwaves and droughts are projected to increase across most of Europe. This trend is already evident between 2000 and 2023, during which eight years recorded above-average drought-affected areas, with five of them within the last decade alone. The recurrence and growing severity of these events over the past 24 years suggest that a decline in drought-impacted areas by 2030 is unlikely^56^. A particularly pressing issue in Poland is the uneven distribution and variability of water resources across both time and space^57^. Recent changes in the atmospheric climate have led to a variety of impacts, among which alterations in the water balance due to a marked increase in field evaporation are the most pronounced. Since 1988, Greater Poland has been increasingly affected by water deficits, posing a direct threat to the supply needs of several key economic sectors essential to the region’s sustainability. The most severe precipitation deficits are observed in the central part of the country, which lies within Poland’s lowland region. As in many other areas of continental Europe, relatively low annual precipitation combined with rising temperatures has further exacerbated these pressures^58^. Drought is an inherent feature of the Polish climate, generally persisting for several weeks and affecting large areas. Its consequences can span multiple sectors. Even when the immediate impacts appear limited in scale or significance, they can be long-lasting and critical across various domains. Owing to its effects on the environment, economy, and society, increasing attention has been directed towards research and practical measures aimed at assessing, monitoring, and forecasting drought^22^.

The threshold method applied to daily hydrographs from thirteen representative catchments in Poland enabled the identification of key characteristics of streamflow droughts over the past 30 years. This approach, which relies on daily flow variations rather than a fixed annual threshold, allows for a more accurate assessment of hydrological conditions, especially in regions with pronounced seasonality where different types of droughts may occur due to precipitation, temperature, or both. A similar method for defining drought periods was adopted by Rivera et al.^59^ and Seneviratnen et al.^60^. The analysis of the variability of regional average indicators in the hydrological years 1993–2022 indicates that drought conditions were particularly severe in 2003, based on SNDD and SCDV for the 70th and 90th percentiles. Studies by Laaha et al.^61^ and Sutanto and Van Lanen^62^ also indicate that 2003 was a pivotal year, recognised as one of the most important drought years in Europe. The average TNDE value for the Q70 threshold and a minimum drought duration of 7 days is 78 for the analysed catchments (Sama River), while MDD ranges from 20.99 to 68.80 days under the same input parameters. The longest low-flow period in 2003, at the Q70 threshold, was observed in the Bawół River, lasting continuously for 201 days (from 23 May to 9 December). In the Sama River, however, the longest low-flow period observed lasted 118 days (from 5 June to 30 September). The average MDD for Q70 and a minimum duration of 7 days is 42 days, whereas for a minimum duration of 30 days it increases to 77 days. The study by Sutanto and Van Lanen^62^ states that minor droughts are the cause of frequent occurrences of threshold-based droughts, both with variable thresholds (VTD) and fixed thresholds (FTD), in European rivers.

In this study, MDD does not exceed 100 days at Q95 with the duration of the low flow being at least 30 days. By contrast, research by Tomaszewski and Kubiak-Wójcicka^23^ indicated that the average low-flow duration, at a 95% probability of non-exceedance, in Polish rivers is 162 days. The spatial variation of this parameter generally corresponds to the distribution of water resources. Their study analyzed low-flow conditions at 17 gauging stations across Poland between 1951 and 2015 and showed that, in most cases, the maximum duration did not exceed 250 days, although in some cases it exceeded 500 days^23^. Research on the number of low-flow days (NDLF) and long-term changes in river discharge was conducted by Wrzesiński et al.^63^, who analysed flows from 140 gauging stations located on 96 rivers across Poland for the period 1951–2020, as well as for two sub-periods: 1951–1988 and 1988–2020, representing the periods before and after climate change. Their study revealed changes in river discharge, with most gauging stations recording a decrease of approximately 5–15% (in some cases statistically significant). Furthermore, over the entire study period, statistically significant declining trends in NDLF were evident in the eastern and southern parts of the Vistula basin. After 1988, however, this pattern shifted, with NDLF showing an increasing trend, except for a few rivers in southern Poland, where statistically significant decreases were still observed.

The dynamics of low-flow episode progression are characterized by high seasonal and multiannual variability^23^.

In the present study, a seasonal analysis of drought in the rivers under investigation revealed a high incidence of the phenomenon during summer and autumn. At the Q70 threshold, the mean SNDD reached 53 days in summer and 34 days in autumn. Other authors also highlight that the autumn and summer months are the main periods determining severe hydrological drought. According to Tomaszewski and Kozek^64^, severe and extensive hydrological droughts usually occur during summer and early autumn (July–October), with June serving as a transitional month that can markedly prolong drought duration in years characterised by dry springs and summers. Moreover, in the majority of both upland and lowland rivers, the peak period of hydrological drought typically falls in August. Somorowska^25^, on the other hand, notes that droughts of varying intensity occur in Poland during both the winter and summer halves of the year. Her analysis indicates that the most extreme drought occurred in August 2015, lasting three months and affecting 47% of the country’s area.

Given that these catchments span diverse geographical regions (mountains, lowlands, and coastal areas), it can be suggested that extremely severe hydrological droughts are influenced not only by hydrometeorological conditions but also by local catchment-specific factors, arising from a combination of natural processes and human activities^65^. In recent years, land cover in catchments has undergone noteworthy changes both in Poland and globally. These transformations influence various catchment processes, including water retention and river flow. Changes in land cover are driven by multiple environmental factors, such as water circulation, landscape quality, ecosystems^66^, and the hydrological regime^67^. Urbanisation alters catchments through the expansion of impermeable surfaces and modifications to drainage systems, which accelerate runoff and limit infiltration. This disruption of the hydrological cycle not only reduces groundwater recharge but also increases flood risk. The regional hydrological cycle, a key factor in flood management, has been significantly affected by both rising urbanisation and climate change, potentially leading to more extreme precipitation events and severe hydrological disasters in the near future^68^. An increase in impervious surface area reduces the retention capacity of catchments and contributes to local temperature rise^69^. Several studies also indicate that deforestation-related land cover changes are linked to an increased frequency of hydrological extremes^70^^,^^71^. For instance, rapid changes in the Prądnik catchment resulted in a 75% increase in water loss^72^. Intensifying catchment transformations contribute to the increasing severity of droughts, underscoring the importance of forecasting their future development and implementing effective mitigation strategies. There is an urgent need for sustainable water management solutions that preserve natural retention capacity, protect ecosystems, and support climate change adaptation. In this context, analysing long-term memory in hydrological processes using the Hurst exponent proves to be a valuable approach, as it enables assessment of trend persistence and potential future changes in runoff regime and water resources^43,44^. In the analysed area, the Hurst exponent for all studied rivers exceeded 0.5, with an average value of 0.78. This indicates the persistence of current hydrological trends and highlights the index’s value in strategic planning and water resource management. In their study, Millen and Beard^72^ applied the Hurst exponent to the Burdekin River in Australia, where the estimated value was 0.7527. As with the rivers analysed in this study, a value above 0.5 indicated that all hydrological variables are likely to follow persistent trends in the future. Tatli^73^ also used the Hurst exponent to examine drought conditions in Turkey, observing values close to 1 in areas vulnerable to future droughts. These findings support the assumption that long-range memory in large-scale climate systems and/or teleconnections plays a key role in explaining drought occurrence. Given that global warming is one of the major aspects of climate change, it further intensifies drought conditions. Similarly, studies conducted in Mongolia confirmed the reliability of the Hurst exponent in predicting drought trends, with a forecasting accuracy of up to 91.7%. In that study, the average Hurst value of the SPEI time series from 1980 to 2014 was 0.533, indicating that future drought trends are generally consistent with the present state^74^. The Hurst exponent can be a highly practical indicator for informing water and drought management policies. It has also been applied in flood risk prediction, demonstrating its versatility in hydrological assessments. To address the increasing challenge of hydrological drought, it is crucial to implement adaptive measures tailored to the specific characteristics of each region. This approach is essential for mitigating water deficits, preventing soil degradation, and ensuring reliable water availability for agriculture. Simultaneously, strengthening regional capacity and raising public awareness of climate change impacts on water resources are vital. Only comprehensive, locally adapted management of limited water resources can ensure their long-term and sustainable use^73^.

The results of this study should be interpreted with caution, as they primarily reflect conditions in lowland rivers and may not be directly applicable to mountainous catchments. Future research could extend the analysis to include additional catchments and longer time series, which would allow for a more comprehensive understanding of regional drought patterns.

## Methods

The study area encompasses 15 rivers situated in Poland’s lowland regions (Fig. 1), with catchment areas ranging from 176.01 km² to 726.28 km². These predominantly agricultural regions are highly dependent on sufficient water availability. Daily discharge data were obtained from 15 gauging stations, sourced from the hydrological database of the Institute of Meteorology and Water Management (IMGW). Figure 1 illustrates the spatial distribution of the selected gauging stations along with major Polish rivers. The 15 time series were selected based on data quality, spatial coverage, and the duration of available records. Table 1 lists the gauging stations and provides key details for each.

### Spatial data

The boundaries of river basins and catchments, as well as vector layers of rivers, were obtained from the IIaPGW database (Second Update of River Basin Management Plans), available at https://apgw.gov.pl/. Vector data on land use for the years 2000 and 2018 were sourced from the Copernicus Land Monitoring Service (https://land.copernicus.eu). Vector data on soil types were obtained from the Harmonized World Soil Database (HWSD), version 2.0, available at https://data.isric.org/.

### Data analysis

Daily flow data from 15 hydrological gauging stations, spanning a 30-year period (hydrological years 1993–2022), were analysed using statistical methods, specifically the MK test and Sen’s slope estimator. A 30-year period was selected as it represents the minimum standard recommended by the World Meteorological Organization (WMO) for calculating hydrological drought characteristics and for comparing hydrological data^75^. The MK test is a non-parametric, rank-based test used to detect monotonic trends within time series data. Given the often skewed distributions of hydrometeorological data, the MK test is particularly well suited for this purpose, as is the Sen’s slope estimator. Sen’s slope estimator was used to quantify the magnitude of the trend detected by the MK test, allowing for an assessment of the rate at which a given variable, such as river discharge, changes over the 30-year period. Failure to meet the significance of the MK test (*p* ≤ 0.05), together with the absence of a decreasing trend in the analysed flows, excludes the gauging station from further analyses. Both Sen’s slope and the MK test were performed in the R programming environment.

### Streamflow drought definition

To identify streamflow drought events, we applied the threshold approach, originally proposed by Yevjevich^45^ and widely adopted in subsequent hydrological studies^23,31,60,61,64^. Threshold levels, also referred to as truncation levels, were derived from Severity-Duration-Frequency (SDF) curves at flow values equaled or exceeded 70%, 90%, and 95% (Q70, Q90, and Q95) of the time (≥ 7, ≥ 10, ≥ 20, and ≥ 30 days) respectively, calculated based on daily flow variations (For example SDF curves, see Supplementary Fig. S4 online). These thresholds represent normal, severe, and extreme water flow drought conditions, respectively. This approach enables the identification of both short-term and multi-year droughts^4^.

A key advantage of this method, compared to standardised drought indices, is its ability to quantify deficit magnitude, which is an important factor for water resource management^31^. In this framework, a drought event begins when water flow falls below the threshold and ends when it rises above it.

Hydrological indices, such as TNDE, MDD, MCDV, were calculated for different variants in the R programming environment, using the hydroTSM, remotes, dplyr packages. Subsequently, they were subjected to correlation analysis and visualised as a heat map combined with hierarchical clustering.

To examine the interrelationships among the hydrological indicators (TNDE, MCDV, MDD) and land use changes, principal component analysis (PCA) was applied across all study catchments. Subsequently, PCA was also employed to investigate the association between hydrological indicators and land use. This analysis was conducted for two reference years, 2000 and 2018, across the entire set of catchments. Changes in LUC shares are expressed in pp. All graphs of PCA were created using the ggplot package in R studio.

To show the seasonal variability of low-flow events, the SNDD, SNDE, and SCDV values ​​were calculated in the R programming environment using the dplyr and lubridate packages. Graphs in R were created using the ggplot package.

Based on 30 years of daily data, the Hurst exponent was calculated to predict the future drought trend in the analysed catchments. The Hurst exponent was used to characterise the long-term memory of a time series, providing an indication of the persistence of prevailing trends or patterns. A Hurst exponent greater than 0.5 indicates strong persistence, whereas a value below 0.5 indicates anti-persistence^76^. The Hurst Index was calculated using the hursexp() function in the pracma R package.

## Conclusions

The analysis of hydrological droughts in lowland catchments of Central Poland during the hydrological years 1993–2022 revealed significant changes in flow regimes (*p* < 0.001). Thirteen out of fifteen analysed rivers exhibited a decreasing trend in streamflow, indicating an intensifying water deficit in the studied region. The most pronounced drought occurrences were observed in the catchments of the Skora, Ślęza, and Prosna rivers, where the number of events exceeded 100 over the 30-year period. Land use changes, particularly the increase in anthropogenic areas at the expense of agricultural and forest lands, have a notable impact on the intensification of short-term drought episodes. This phenomenon was particularly evident in the Skora River catchment. The seasonality analysis confirmed that drought events are concentrated in the summer and autumn months, posing a major challenge for water resource management, especially in agriculture and the protection of aquatic ecosystems. Although long-duration droughts (≥ 20 and ≥ 30 days) occurred relatively infrequently, their characteristics, especially the high volume of MCDV, suggest increasing intensity and potentially longer periods of extreme drought events. High Hurst exponent values for the analysed rivers (0.68–0.87) indicate strong autocorrelation and persistence of observed trends, suggesting that unfavorable hydrological conditions are likely to persist in the future. The findings underscore the urgent need for integrated water resource management strategies at the local level, with particular attention to areas most vulnerable to drought, as small rivers play a crucial role in water retention and in maintaining the overall water balance of entire river basins. This methodological approach not only provides valuable insights into drought occurrence but also offers a transferable framework for similar hydrological assessments in other regions. In light of the observed changes, continued research is essential to better understand the impacts of climate and land use transformations on the hydrology of small catchments and their contribution to the functioning of entire river systems.

## Supplementary Information

Below is the link to the electronic supplementary material.

Supplementary Material 1

## Data Availability

All data are publicly available from the Institute of Meteorology and Water Management – National Research Institute (IMGW-PIB) in Poland, via the website https://danepubliczne.imgw.pl/or can be requested by contacting the corresponding author, Ewelina Janicka-Kubiak.
