# Supplementary material for: Hydrological drought trends and seasonality in selected Polish catchments between 1993 and 2022 using a threshold based approach
Source: Sci Rep. 2025 Nov 18;15:40454. doi: 10.1038/s41598-025-24133-1 (PMC12627574; doi:10.1038/s41598-025-24133-1)
Supplement: Supplementary file 1 — Supplementary Material 1 [file 41598_2025_24133_MOESM1_ESM.pdf]

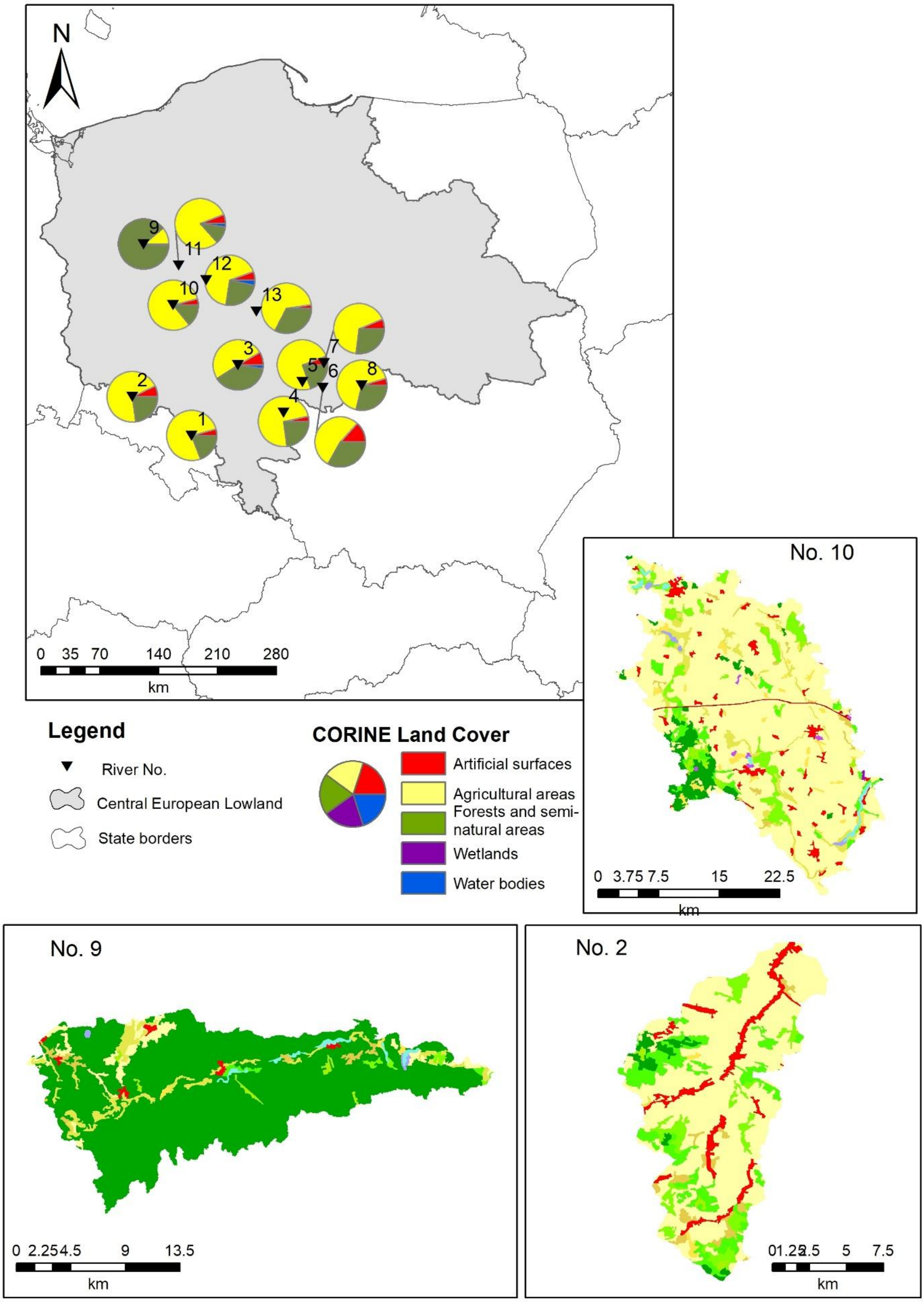

Supplementary Figure 1. Land use (LU) in catchments in 2018.

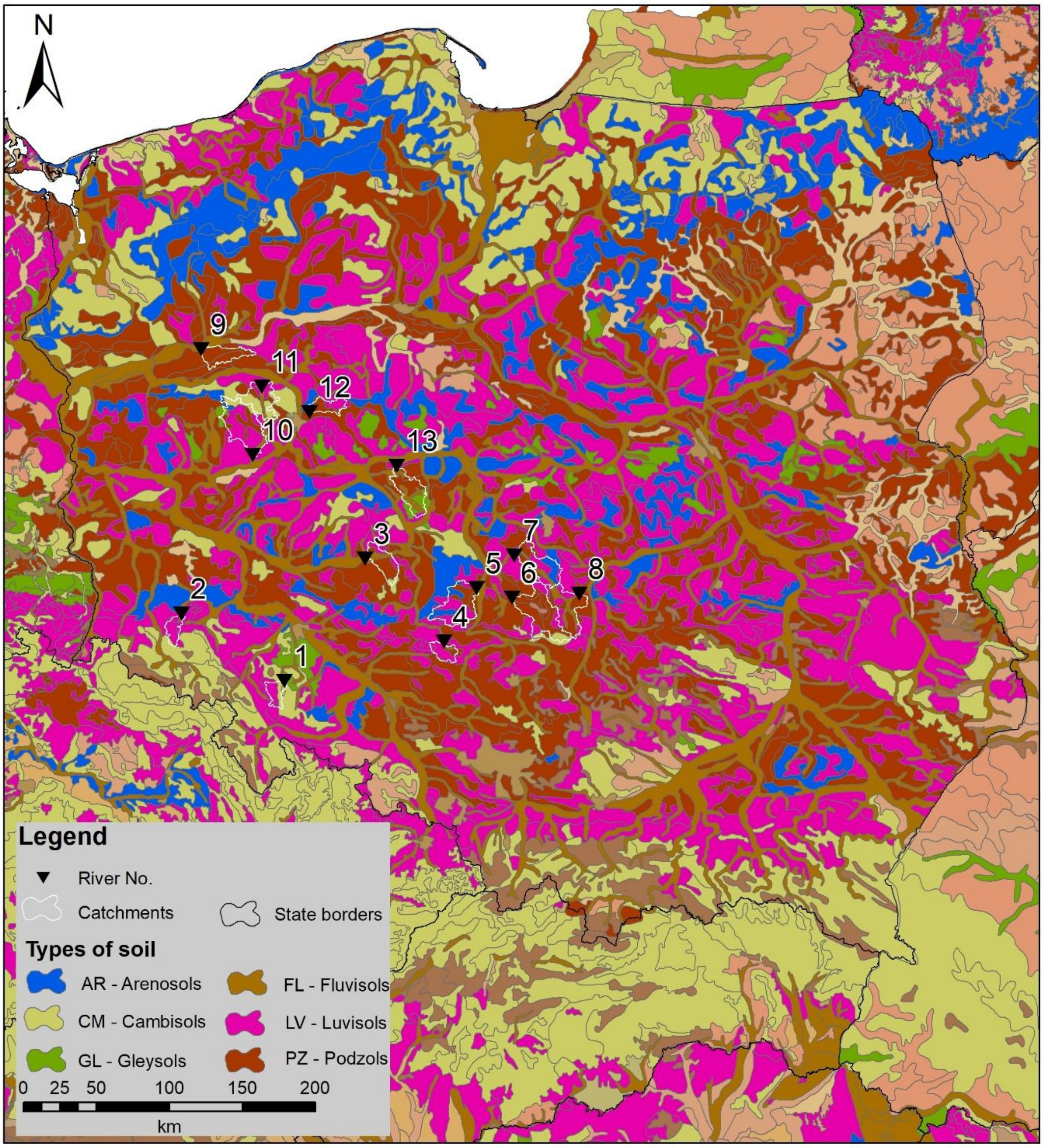

Supplementary Figure 2. Soil types for selected catchments.

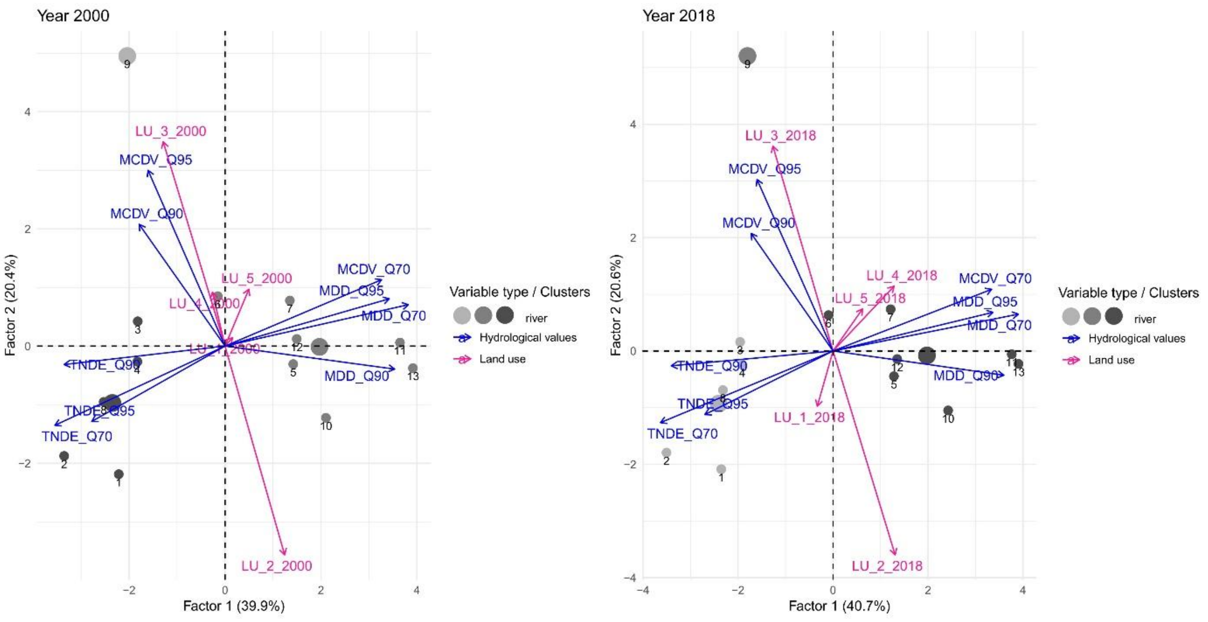

Supplementary Figure 3. Principal Component Analysis (PCA) showing hydrological parameter values and land use, and rivers, with clusters for 2000 and 2018.

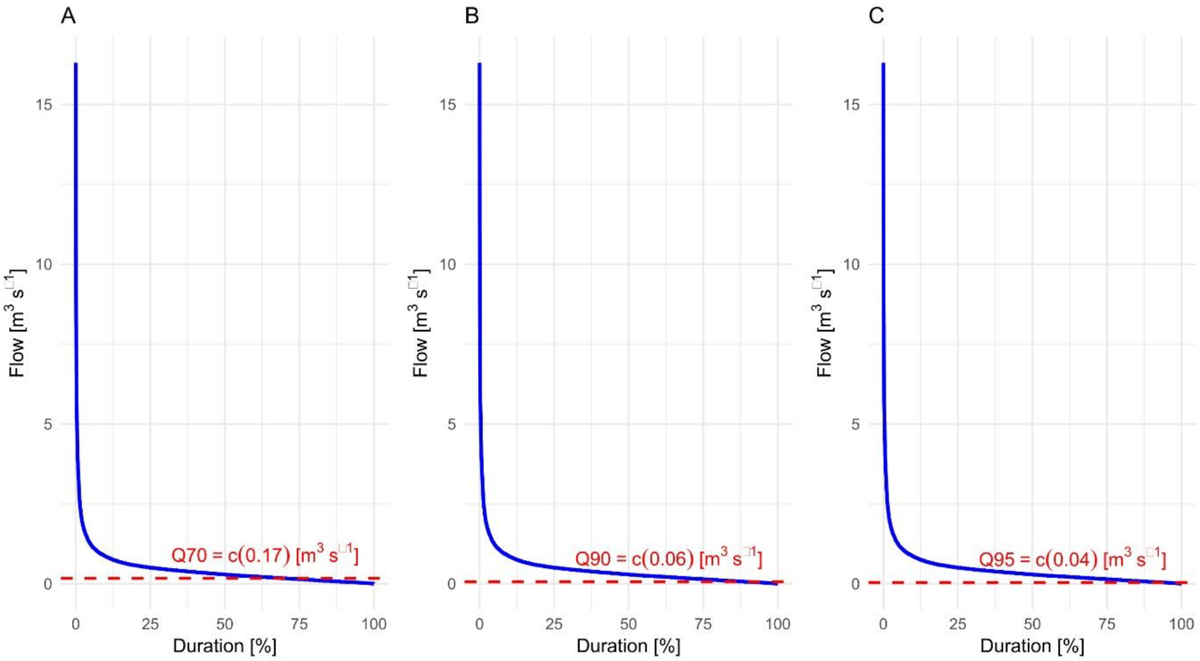

Supplementary Figure 4. The Severity-Duration-Frequency (SDF) curve for the Ślęza River at thresholds (A) Q70, (B) Q90, and (C) Q95.
